# A ruthenium polypyridyl intercalator stalls DNA replication forks, radiosensitizes human cancer cells and is enhanced by Chk1 inhibition

**DOI:** 10.1038/srep31973

**Published:** 2016-08-25

**Authors:** Martin R. Gill, Siti Norain Harun, Swagata Halder, Ramon A. Boghozian, Kristijan Ramadan, Haslina Ahmad, Katherine A. Vallis

**Affiliations:** 1 CRUK/MRC Oxford Institute for Radiation Oncology, Department of Oncology, University of Oxford, Oxford, UK; 2 Department of Chemistry, Faculty of Science, Universiti Putra Malaysia, Malaysia

## Abstract

Ruthenium(II) polypyridyl complexes can intercalate DNA with high affinity and prevent cell proliferation; however, the direct impact of ruthenium-based intercalation on cellular DNA replication remains unknown. Here we show the multi-intercalator [Ru(dppz)_2_(PIP)]^2+^ (dppz = dipyridophenazine, PIP = 2-(phenyl)imidazo[4,5-f][1,10]phenanthroline) immediately stalls replication fork progression in HeLa human cervical cancer cells. In response to this replication blockade, the DNA damage response (DDR) cell signalling network is activated, with checkpoint kinase 1 (Chk1) activation indicating prolonged replication-associated DNA damage, and cell proliferation is inhibited by G1-S cell-cycle arrest. Co-incubation with a Chk1 inhibitor achieves synergistic apoptosis in cancer cells, with a significant increase in phospho(Ser139) histone H2AX (γ-H2AX) levels and foci indicating increased conversion of stalled replication forks to double-strand breaks (DSBs). Normal human epithelial cells remain unaffected by this concurrent treatment. Furthermore, pre-treatment of HeLa cells with [Ru(dppz)_2_(PIP)]^2+^ before external beam ionising radiation results in a supra-additive decrease in cell survival accompanied by increased γ-H2AX expression, indicating the compound functions as a radiosensitizer. Together, these results indicate ruthenium-based intercalation can block replication fork progression and demonstrate how these DNA-binding agents may be combined with DDR inhibitors or ionising radiation to achieve more efficient cancer cell killing.

Upon origin firing during S phase of the cell-cycle, the formation and progression of stable replication forks allows the faithful duplication of the genome and is essential for mammalian cell proliferation^1^. Accordingly, small molecules that stall replication forks such as hydroxyurea (HU) and camptothecin (CPT) have proven invaluable in the elucidation of the molecular biology of DNA replication in human cells^2^,^3^,^4^. Furthermore, due to the high rate of cancer cell proliferation compared to normal cells, drugs able to inhibit DNA synthesis are used to treat cancer, often concurrently with radiotherapy^5^. Examples include cisplatin (cis-diamminedichloroplatinum(II)), a reactive platinum(II) complex that generates inter- and intra-strand platinum-DNA crosslinks that block replication^6^, and gemcitabine (2′,2′-difluorodeoxycytidine), a nucleoside analogue that blocks DNA synthesis through incorporation into extending DNA strands^7^. Other drugs stall replication forks by reversible (i.e. non-covalent) binding interactions. These include doxorubicin (DOX), a DNA intercalator and topoisomerase II poison that generates trapped topoisomerase cleavage complexes that present a physical barrier to the moving fork^8^. However, use of these DNA-damaging agents is limited by their high toxicity and acquired or intrinsic drug-resistance. Thus, there remains a need to develop compounds that inhibit cancer cell proliferation by novel mechanisms of action, with reduced adverse effects on healthy cells and that can be combined safely with radiation therapy.

Over the last three decades, the DNA-binding properties of ruthenium(II) polypyridyl coordination or organometallic complexes (RPCs) have been the focus of intense study^9^,^10^. As RPCs possess octahedral molecular geometries unobtainable to traditional carbon-based pharmacophores, unique biomolecular binding interactions may be achieved^11^. Furthermore, as many complexes are phosphorescent^12^, they possess a dual imaging capacity that allows verification of intracellular DNA targeting^13^,^14^. While the majority of ruthenium-based anticancer compounds owe their effects to their reactivity and formation of coordinate (irreversible) bonds with DNA in a similar manner to cisplatin^15^, there has been growing interest in the bioactivity of RPCs that bind DNA solely by intercalation^9^. Although several RPC metallo-intercalators have been shown to inhibit cancer cell proliferation *in vitro* and *in vivo*^16^,^17^,^18^,^19^,^20^,^21^, there are surprisingly few examples implying DNA binding is responsible for bioactivity^22^,^23^. Moreover, the impact of RPC intercalation on DNA replication and the cellular response to induced DNA damage are completely unexplored. Considering the detailed knowledge of RPC binding interactions with isolated DNA^24^,^25^,^26^,^27^, and how cellular mechanistic understanding can provide new therapeutic opportunities for DNA-targeting anticancer drugs^28^,^29^, this deficiency is a clear barrier in the development of RPCs towards biological – and potential clinical - application.

In this study, the novel RPC intercalators **1** and **2** (Fig. 1a) were synthesised, where each complex contains three intercalating ligands coordinated to the central Ru(II) ion, and their effects in human cancer cells were characterised in detail. Remarkably, we find complex **1** stalls DNA replication forks in HeLa cervical cancer cells immediately upon addition; an unprecedented finding for a pure metallo-intercalator. We utilise this mechanistic insight to demonstrate the combination of **1** and a pathway-specific DNA damage response (DDR) inhibitor achieves synergistic cell death specifically in cancer cells. Furthermore, we show treatment with **1** sensitises cancer cells to the effects of external beam ionising radiation, indicating the complex is an efficient radiosensitizer. These discoveries indicate that ruthenium polypyridyl complexes are useful tools to probe the molecular consequences of DNA intercalation and also strengthen the case that this class of compounds merit further investigation as anticancer drugs, especially within combinational therapy roles.

## Results

### DNA binding

**Figure 1:**
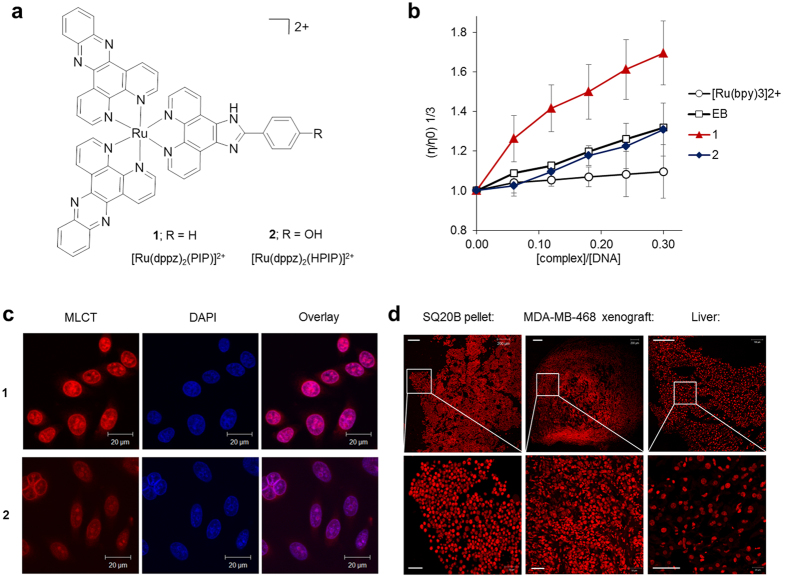
Ruthenium(II) polypyridyl complexes 1 and 2 intercalate DNA. (**a**) Chemical structures of **1** and **2**. (**b**) Increasing relative viscosity (η/η_0_) of calf thymus DNA (4.19 × 10^−5 ^M) with the addition of **1** or **2**. Data are the mean of two independent experiments +/− SD. Intercalator ethidium bromide (EB) and non-intercalating [Ru(bpy)_3_]^2+^ included for comparison. (**c**) PFA-fixed HeLa cells stained with either **1** or **2** (100 μM, 10 mins) showing co-localisation of MLCT (metal to ligand charge-transfer) emission with nuclear DNA dye DAPI. See SI for single-dye control micrographs. (**d**) Paraffin-embedded pellet (SQ20B cells) or frozen tissue (MDA-MB-468 tumour xenograft and normal liver) sections stained with **1** (5 mM, 1 h) showing nuclear localisation (bottom panels) visualised by CLSM. Top row scale bars = 200 μm. Bottom row scale bars = 50 μm.

The novel ruthenium(II) polypyridyl complexes [Ru(dppz)_2_(PIP)]^2+^, **1**, and [Ru(dppz)_2_(*p*-HPIP)]^2+^, **2**, (dppz = dipyrido[3,2-a:2′,3′-c]phenazine, PIP = (2-(phenyl)imidazo[4,5-f][1,10]phenanthroline) and *p*-HPIP ((2-(4-hydroxyphenyl)imidazo[4,5-f][1,10]phenanthroline)) were prepared by modification of reported synthetic pathways^17^,^30^,^31^ and characterised by standard spectroscopic and analytical techniques (Figs S1–7 in the Supplementary Information). Evidence of a reversible binding interaction with DNA is provided by evidence of hypochromicity of the absorption spectra of **1** and **2** with increasing concentrations of DNA (Fig. S8). While resultant Scatchard plots from the derived ligand:DNA binding curves did not fit the McGhee von Hippel model, likely due to the existence of multiple binding interactions, applying a simple binding model^30^ in its place resulted in the estimation of binding constants, K_b_, of 2.5 × 10^6^ M^−1^ and 6.7 × 10^7^ M^−1^ for **1** and **2** respectively, indicating each complex binds DNA with high affinity (Table 1). To investigate specific binding mode, relative viscosities of DNA with increasing concentrations of each complex were determined, where an increase in relative viscosity corresponds to the unwinding and lengthening of the DNA strand as a result of intercalation^32^. Figure 1b shows complex **1** and **2** both increase relative viscosity of DNA, confirming that both these compounds bind DNA by intercalation. Notably, the increase in relative viscosity of DNA on addition of **1** was significantly greater than that for either **2** or ethidium bromide (EB), indicating **1** induces structural distortion to DNA at a substantially greater level than mono-intercalation, and is therefore consistent with a multi-intercalative binding interaction^33^. In addition to these cell-free DNA binding studies, the phosphorescent metal to ligand charge-transfer (MLCT) excitation/emission wavelengths may provide a direct indication of cellular DNA binding and several RPCs function as DNA imaging probes^13^,^14^. Accordingly, the ability of **1** and **2** to visualise DNA in fixed and membrane-permeabilised cells was characterised employing CLSM (confocal laser scanning microscopy). Using this method, HeLa cell nuclei were imaged successfully using each complex and marked co-localisation with the DNA dye DAPI (4′,6-diamidino-2-phenylindole) was observed (Figs 1c and S9). Furthermore, we also show complex **1** allows visualisation of intracellular components of a paraffin-embedded cell pellet (SQ20B head and neck squamous carcinoma cells) and frozen tissue sections taken from a mouse xenograft model (MDA-MB-468 human breast cancer cells) or normal liver, where nuclear MLCT staining by **1** is evident upon higher magnification (Figs 1d and S10).

### Impact on cell proliferation and cellular internalisation

**Figure 2:**
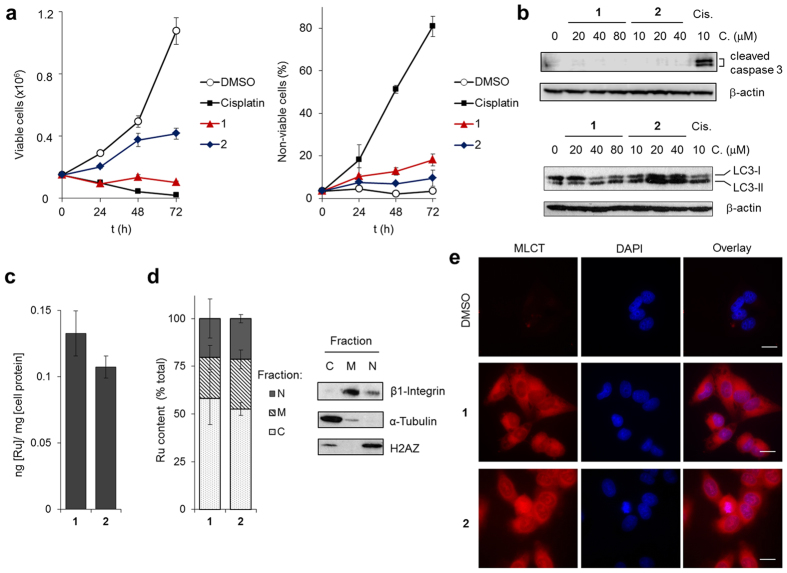
Complexes 1 and 2 are internalised by cancer cells and impact proliferation. (**a**) Effect of 40 μM **1** or **2** (0–72 h incubation time) on numbers of viable (left) and non-viable (right, data expressed as % total cells, independent of viability) HeLa cells (in triplicate, +/− SD). DMSO (0.2%) blank and cisplatin (20 μM) included for comparison. (**b**) Western blotting of lysates from HeLa cells treated with **1**, **2** or cisplatin (24 h) probed for increased levels of apoptosis marker cleaved caspase 3 (upper panels) or autophagy marker LC3-II (lower panels). β-actin was employed as a loading control. Concentration ranges for **1** and **2** were centred on IC_50_ values, Table 1. (**c**) Intracellular Ru levels of HeLa cells treated with **1** or **2** (40 μM, 24 h), as determined by ICP-MS. (**d**) Ru levels within isolated subcellular fractions of HeLa cells treated with **1** or **2** (40 μM, 24 h). Fractions: C = cytoplasm and cytoskeleton, M = membrane, N = nucleus. Successful fractionation confirmed by immunoblotting (right). All ICP-MS data were normalised to protein content of the corresponding fraction and are the mean of two independent experiments +/− SEM. (**e**) Epifluorescence microscopy of HeLa cells treated with **1** or **2** (40 μM, 24 h) showing intracellular MLCT emission. Co-staining with nuclear stain DAPI included. Scale bars = 20 μm.

To provide an indication of anti-proliferative potency of **1** and **2**, we incubated two human cancer cell lines (HeLa cervical and MCF7 breast) and primary human foreskin fibroblasts (HFFs) with a concentration gradient of each complex and assessed the resultant impact on cell viability by the MTT metabolic activity assay (MTT = 4,5-dimethylthiazol-2-yl)-2,5-diphenyltetrazolium bromide) from which half-inhibitory (IC_50_) concentrations were derived. The cytotoxic DNA-damaging anti-cancer drugs cisplatin and mitoxantrone (MX), an organic intercalator and topoisomerase II poison^33^, were included in parallel for comparison. These experiments indicate that both Ru(II) compounds impact cell proliferation, with **2** demonstrating greater potency than **1** towards both cancer cell lines and possessing comparable IC_50_ values to cisplatin (Table 1). Importantly, both **1** and **2** showed minimal activity towards non-malignant HFF cells, with IC_50_ concentrations >100 μM. In contrast, MX shows high activity (IC_50_ < 5 μM) towards *all* cell types, including HFFs, reflecting the non-specific cytotoxicity of this organic intercalator (Table 1). As MTT assays do not discriminate between growth inhibition or cytotoxicity^34^, the ability of **1** and **2** to impact cell growth and/or induce cell death was investigated by Trypan Blue exclusion assay. These results indicated treatment with 40 μM **1** completely halts HeLa cell growth following 24–72 h treatment (Fig. 2a, left). Notably, the levels of non-viable (Trypan Blue positive, i.e. membrane-compromised necrotic cells) populations in cells treated with **1** remain relatively low (<20%), indicating modest cytotoxicity (Fig. 2a, right). Additionally, these results indicated that complex **2** is not as effective as **1** in halting cell growth, despite possessing a greater potency as determined by MTT assay. Examination of specific cell death pathway activation showed no generation of the apoptosis marker cleaved caspase-3^35^ in HeLa cells treated with either **1** or **2** (Fig. 2b, top), behaviour in contrast to the apoptosis-inducing agent cisplatin, and cells treated with **1** showed no detectable increase in levels of the autophagy marker LC3-II^36^ (LC3 = Microtubule-associated protein light chain 3) (Fig. 2b, bottom). However, these results revealed LC3-II levels are greater in cells treated with **2** at IC_50_ concentrations or greater compared to untreated (Fig. 2b). Furthermore, quantifying LC3 levels revealed a distinct increase in the ratio of LC3-II to LC3-I, a hallmark of autophagy induction^36^, in **2**–treated cells from exposure times of 8 h onwards (Fig. S10).

A key aspect of RPC bioactivity are their cellular internalisation properties^9^. Accordingly, the cellular uptake and subcellular localisation of each complex was quantified using inductively coupled plasma mass spectroscopy (ICP-MS). HeLa cells were incubated with **1** or **2** and whole cell lysates or isolated cell fractions (C = cytoplasm and cytoskeleton, M = membrane and N = nuclear) from lysates analysed for total ruthenium content. Analysis of whole cell lysates revealed a greater level of cellular ruthenium content for cells treated with **1** compared to **2** (Fig. 2c), in agreement with the concept that increased RPC hydrophobicity promotes cellular uptake^37^ (Table 1). Analysis of isolated subcellular fractions showed a similar intracellular distribution of **1** and **2**, where the greatest Ru concentration was in the cytosol/cytoskeletal component (58 and 52% for **1** and **2** respectively) and nuclear uptake represented ~20% of total Ru content in cells treated with either **1** or **2** (Fig. 2d). In addition to this, visualising HeLa cells post-treatment with **1** or **2** by wide-field epifluorescence microscopy showed a broad intracellular distribution of each complex, where significant MLCT emission was observed in the cytoplasm alongside a lower contribution localised in the nucleus (Figs 2e and S11).

### [Ru(dppz)_2_(PIP)]^2+^ stalls replication forks

**Figure 3:**
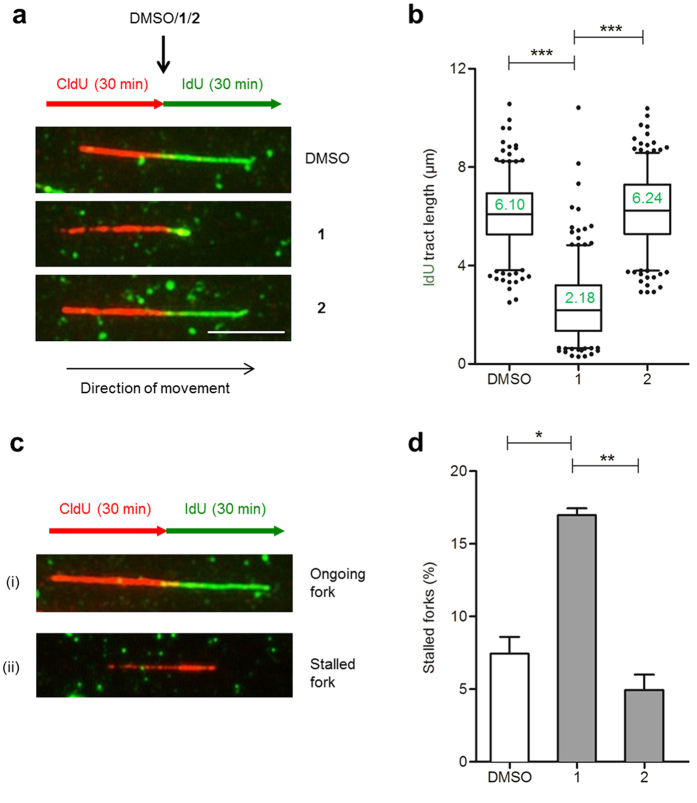
[Ru(dppz)_2_(PIP)]^2+^ stalls replication fork progression in human cancer cells. (**a**) DNA fibres from HeLa cells labelled sequentially with CldU (red) and IdU (green) (30 mins each) where DMSO (control), **1** or **2** (40 μM) were added during the incorporation of IdU (green). Representative replication forks for each treatment condition shown. Scale bar = 5 μm. (**b**) Statistical analysis of IdU tract length in cells treated as in (**a**). Middle line represents median and the box extends from the 25^th^ to 75^th^ percentiles. Whiskers mark the 5^th^ and 95^th^ percentiles. At least 300 tracts were counted per experiment. (**c**) Specific replication events (i–ii). (**d**), Quantification of the frequency of stalled forks (as in Fig. 2c, ii) for cells treated as described in (**a**). Data in b,d were analysed using unpaired two-tailed t test. *P < 0.1, **P < 0.01, ***P < 0.005.

As **1** and **2** intercalate DNA with high affinity, impact cell proliferation and demonstrate evidence of nuclear accumulation, we examined the ability of each complex to interfere with replication by performing DNA fibre analyses. Replication tracks were sequentially pulse-labelled with CldU (red) and IdU (green) and the addition of **1** or **2** during the second (IdU) pulse enabled us to evaluate the immediate effect of each complex on individual replication fork progression in S phase cells (Figs 3a and S12). Remarkably, cells treated with **1** demonstrated a dramatic three-fold shortening of the median IdU tract length (green) compared to mock-treated (median IdU tract lengths of 6.10 and 2.18 μm for mock- and **1**-treated cells respectively), indicating rapid and large-scale fork slowdown and stalling directly upon addition of **1** (Fig. 3b). Furthermore, **1** substantially increased the number of stalled replication forks, defined as red tract only, where 17 +/− 1% of total replication forks were stalled compared to 7.5 +/− 1% in the mock-treated samples (Fig. 3c,d). These results indicate **1** stalls replication fork progression immediately upon addition. Moreover, the substantial three-fold decrease in IdU tract length in cells treated with **1** is a markedly greater replication block than observed in related studies employing traditional DNA synthesis inhibitors, including cisplatin and CPT^4^, and levels of stalled replication forks are greater than seen for DOX treatment^38^; findings that emphasise the pronounced replication block by **1**. In contrast to the results for **1**, complex **2** had negligible impact on replication fork progression, either in terms of replication tract length or the number of stalled forks, with both parameters similar to mock-treated controls (Fig. 3b,d).

### [Ru(dppz)_2_(PIP)]^2+^ activates Chk1 checkpoint kinase and prevents cell-cycle progression

**Figure 4:**
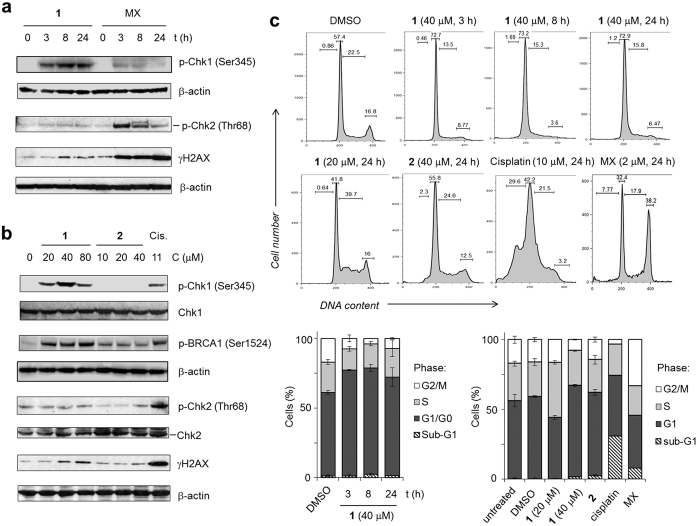
[Ru(dppz)_2_(PIP)]^2+^ induces Chk1 checkpoint kinase activation and G1-S cell-cycle arrest. (**a,b**) Whole-cell extracts of HeLa cells treated with **1** (40 μM) or mitoxantrone (MX, 2 μM) for 3, 8 or 24 h (**a**) or a concentration gradient of **1** or **2** (24 h incubation time, b) were immunoblotted for activated (phosphorylated, p) DNA damage response (DDR) signalling proteins pChk1 (Ser345), pBRCA1 (Ser1524), pChk2 (Thr68) and γH2AX (pH2AX at Ser319), as indicated. β-actin was used as a loading control and cisplatin employed as a positive control for DDR activation. Concentration ranges for **1** and **2** were centred on IC_50_ values, Table 1. See SI for densitometry. (**c**) Cell-cycle distributions of HeLa cells incubated with DMSO (0.2%), **1** or **2** at the stated concentrations and time. DNA content was quantified using propidium iodide (PI) and analysed by flow cytometry. Cisplatin (10 μM) and MX (2 μM) treatment were included for comparison. Data mean of three independent experiments +/− SD.

Induced replication stress and DNA damage activates a network of intracellular signalling pathways known collectively as the DNA damage response (DDR)^29^. The cell-cycle checkpoint kinases Chk1 and Chk2 are essential components of DDR signalling in mammalian cells; Chk1 is phosphorylated on Ser345 sites by ATR (ataxia telangiectasia and Rad3-related protein) as a direct response to stalled replication forks^39^, while Chk2 is phosphorylated by ATM (Ataxia telangiectasia mutated) at Thr68 sites primarily as a result of DSB generation^29^. To characterize DDR activation in response to **1**-stalled replication forks, lysates from treated HeLa cells were prepared and the phosphorylation status of Chk1 (Ser345) or Chk2 (Thr68) determined using phospho-specific antibodies and Western blotting. Cisplatin and MX were used as positive controls for DDR activation and **2** was included for comparative purposes. Treatment of HeLa cells with **1** results in Chk1 phosphorylation from exposure times of 3 h onwards and the levels of phospho-Chk1 were greater than in cells treated with MX or cisplatin (Figs 4a,b and S13). Chk1 pathway activation by **1** is supported by the observation of increased levels of phospho-BRCA1, a protein which mediates Chk1 activation when phosphorylated at Ser1524 sites^40^. Low Chk2 pathway activation by **1** were observed, with levels of phosphorylated Chk2 protein remaining comparable to negative controls (Fig. 4a,b). Consistent with this, relatively low levels of the DSB-marker γH2AX (p-H2AX at Ser139)^41^ occur in lysates of cells treated with **1** (Figs 4a,b and S13). As expected^8^,^42^, substantial Chk2 phosphorylation and γH2AX expression was observed in cells treated with the DSB-generating agents MX or cisplatin. In comparison to the cellular response to **1**, treatment with **2** resulted in lower levels of phosphorylated DDR proteins, indicating a reduced DDR activation in cells exposed to the hydroxyl-substituted complex (Figs 4b and S13).

Examining the impact of **1** on cell-cycle progression, exposure of cycling HeLa cells to 40 μM **1** resulted in an increase in the proportion of cells with G1 phase DNA content and a corresponding decrease in cells in G2/M phases compared to the DMSO control (Fig. 4c). This perturbation of the cell-cycle is observed after 3 h exposure to **1** and remains unchanged with increasing exposure time, indicating rapid and profound cell-cycle deregulation. Furthermore, treating cells with a sub-IC_50_ concentration of **1** (20 μM) resulted in a significant increase in S phase content (Fig. 4c). Cells treated with **2** showed no change in cell-cycle distribution compared to controls. As expected^6^,^8^, MX treatment resulted in a distinct G2 phase increase while cisplatin treatment results in a large decrease in G2/M content as cells arrest at the G1/S transition and progression is slowed through S phase. Notably, cisplatin treatment additionally results in a large percentage (~30%) of cells with sub-G1 DNA content, confirming cisplatin-induced apoptosis. In contrast, only a small increase in sub-G1 population was observed in **1**-treated cells compared to DMSO control, in agreement with the observation that **1** does not induce high levels of apoptosis (*vide supra*). Combined with the relatively low levels of cell death observed in response to **1** (Fig. 2), these findings indicate **1** inhibits cell growth primarily by activating the G1/S and intra-S DNA damage checkpoints thus preventing cell-cycle progression.

### Combination of [Ru(dppz)_2_(PIP)]^2+^ and a Chk1 inhibitor results in synergistic apoptosis in cancer cells

**Figure 5:**
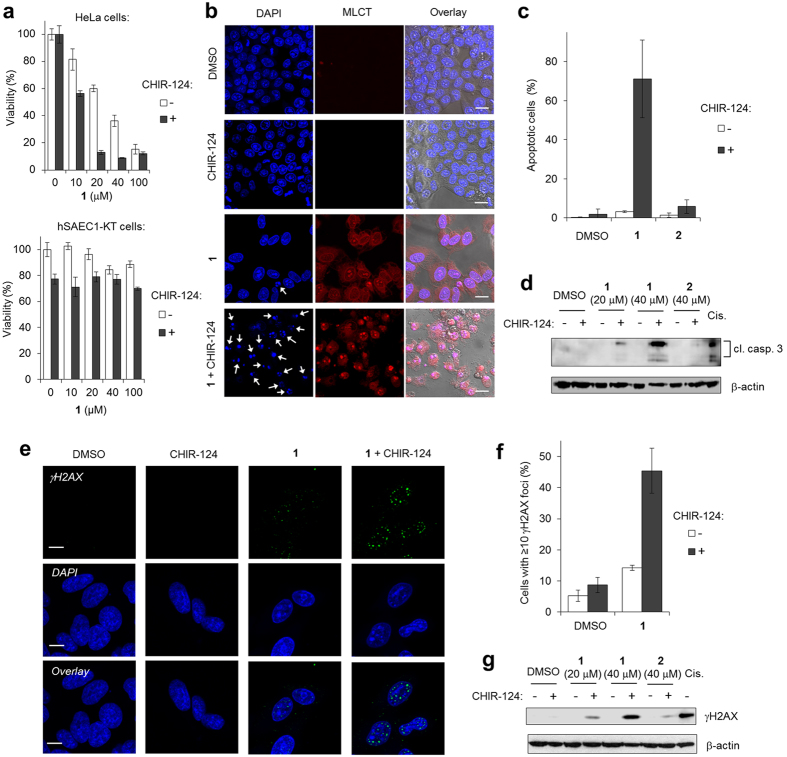
Concurrent treatment of [Ru(dppz)_2_(PIP)]^2+^ plus the Chk1 inhibitor CHIR-124 potentiates synergistic apoptosis in cancer cells. (**a**) Viability of HeLa (cervical cancer) or hSAEC1-KT (normal epithelial) cells after incubation with **1** in the absence or presence of Chk1 inhibitor CHIR-124 (500 nM) for 48 h constant exposure. (**b**) Morphological evidence for apoptosis (pyknosis/karyorrhexis, arrows) in HeLa cells treated with 40 μM **1** plus CHIR-124 for 48 h. Scale bars = 20 μm. (**c**) Quantification of apoptotic cells for experimental conditions as shown in (**b**). Equivalent results for **2** (40 μM, 48 h) included. Data mean of two independent experiments +/− SD. (**d**) Immunoblot analysis for cleaved (cl.) caspase 3 levels (19 and 17 kDa fragments indicated) in HeLa cells treated as in (**b**) and (**c**). Cisplatin incubation (20 μM, 24 h) employed as a positive control and β-actin levels were monitored as a loading control. (**e**) γH2AX foci (green) in HeLa cell nuclei after treatment with 40 μM **1** +/− CHIR-124 for 24 h. Nuclear staining (DAPI) included (blue). Scale bars = 10 μm. (**f**) Quantification of γH2AX foci from cells treated as in (**e**). Data mean of two independent experiments +/− SEM. Minimum of 150 cells counted per experiment. (**g**) Western blot analysis for γH2AX levels in HeLa cells treated as in (**e,f**). Cisplatin treatment (20 μM, 24 h) was included as a positive control for γH2AX induction. β-actin levels were monitored as a loading control.

Based on the principle that many cancers possess high rates of proliferation combined with inherent defects in their DDR signalling pathways^28^, checkpoint kinase inhibition is viewed as a promising strategy to potentiate the efficiency of DNA-damaging drugs^43^. With this in mind, and considering the strong Chk1 activation in response to **1**, HeLa cervical cancer cells, which are p53-mutant^44^, were exposed to a combination of **1** plus the Chk1 inhibitor CHIR-124^45^ and the impact of co-treatment upon cell viability determined. A non-cytotoxic dose of 500 nM CHIR-124 was used and results were compared to single-agent treatment. After 48 h exposure, simultaneous treatment of HeLa cells with **1** and CHIR-124 resulted in a significant decrease in cell viability compared to either agent alone (Fig. 5a, top). Encouragingly, no substantial impact on cell health was observed towards normal hSAEC1-KT human epithelial cells at any combination of **1** and CHIR-124, even at high concentrations of **1** (Fig. 5a, bottom). Flow cytometric data indicated that **1**-induced G1-S cell-cycle arrest in HeLa cells was abrogated by treatment with CHIR-124 (Fig. S14), consistent with its role as a checkpoint inhibitor and confirming **1** induces Chk1-mediated cell-cycle arrest. Minimal enhancement of the anti-proliferative effect of **2** by CHIR-124 was observed (Fig. S15b), in agreement with **2** operating via a Chk1-independent mechanism. Close examination of HeLa cells co-treated with **1** plus CHIR-124 revealed the vast majority of cells demonstrated late apoptotic morphology with high levels of pyknosis and/or karyorrhexis (Fig. 5b,c) accompanied by substantial cleaved caspase 3 expression (Fig. 5d). Single-agent treatment with either **1** or CHIR-124 resulted in minimal morphological evidence of apoptosis (<5%) and no observable cleaved caspase 3 generation (Fig. 5c,d), indicating a synergistic induction of apoptotic cell death as a result of co-treatment. As expected, cells treated with **2 **+ CHIR-124 showed only a low increase in pyknosis/karyorrhexis and cleaved caspase 3 expression remained comparable to single-agent treatment conditions (Figs 5c,d and S15c).

In human cells, activated Chk1 acts to stabilise stalled replication forks, preventing collapse and DSB generation^46^. Therefore, the level of DSB formation in Chk1-comprimised cells was investigated by evaluating γH2AX expression in concomitant treatment conditions. As γH2AX levels are also increased as a result of DNA fragmentation during apoptosis^47^, a reduced incubation time of 24 h was used to examine γH2AX levels at a largely pre-apoptotic exposure, as confirmed by nuclear morphology (Fig. S15c). At this earlier timepoint, a significant increase in the number of γH2AX foci in cells co-treated with **1** and CHIR-124 for 24 h was apparent, indicating an increase in DSB formation compared to single-agent treatment (Fig. 5e,f). This was confirmed examining levels of γH2AX expression by Western blot, where a substantial increase in γH2AX levels were seen in cells co-treated with 40 μM **1** plus CHIR-124 compared to single-agent treatment (Fig. 5g). These findings are consistent with the concept that Chk1 inhibition facilitates the conversion of stalled replication forks to DSBs, the increased levels of which trigger apoptosis.

### [Ru(dppz)_2_(PIP)]^2+^ functions as a radiosensitizer in human cervical cancer cells

**Figure 6:**
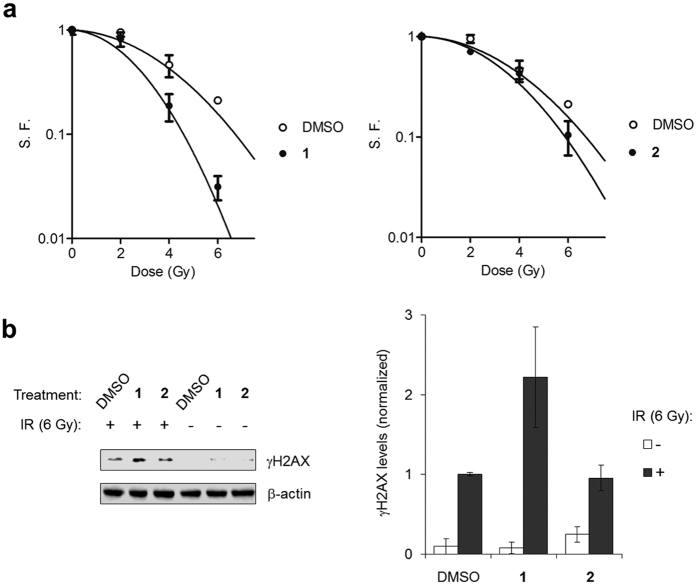
[Ru(dppz)_2_(PIP)]^2+^ sensitises human cancer cells to external beam ionising radiation. (**a**) HeLa cells were incubated in the absence (⚪) or presence (⚫) of 40 μM **1** or **2** for 20 h prior to irradiation with 0–6 Gy ^137^Cs-γ-rays and 4 h after. Survival fraction (S. F.) as a result of each treatment was determined by clonogenic assay and is presented as the mean +/− SD of two independent experiments where each experiment was performed in triplicate. (**b**) Expression of γH2AX in cells treated with **1** or **2** (40 μM) for 20 h followed by 6 Gy IR. Cells were harvested 1 h after irradiation and lysates analysed by Western blotting (left). γH2AX levels were measured by densitometry relative to β-actin loading controls and normalised to DMSO + 6 Gy data (right). Data average of two independent experiments +/− S.E.M.

Chemotherapeutics are often used in combination with external beam ionising radiation in the treatment of cancers and many drugs that inhibit DNA replication are potent radiosensitizers^5^,^48^. To examine the possibility that **1** may function as a radiosensitizer, HeLa cells were pre-treated with **1** for 20 h before irradiation (0–6 Gy ionising irradiation (IR); ^137^Cs-γ-rays; dose rate = 0.809 Gy min^−1^). After further incubation for 4 h with the complex, the relative impact on cell viability was assessed by clonogenic survival. Complex **2**, which achieves its impact on cell proliferation through non-DNA damage-based autophagy, was included in parallel for comparison. Figure 6a shows cells treated with 40 μM **1 **demonstrate an enhanced sensitivity to IR, where the survival of combined treatment was decreased compared to radiation alone: For example, the combination of **1** and 6 Gy γ-rays results in a survival fraction (S.F.) of 3.1 +/− 1% compared to 21.1 +/− 2% for cells treated with 6 Gy γ-rays alone; a 6.8 fold decrease in cell survival. The S. F. of HeLa cells treated with **1** in the absence of radiation was ~85%, indicating that essentially non-lethal concentrations of **1** result in supra-additive radiation cytotoxicity. The radiation enhancement ratio (RER) for cells treated with **1** compared to mock-treated was calculated to be 1.48 (Table S2), a radiosensitizing factor comparable to gemcitabine in the same cell line^48^. While a relatively high concentration of **1** is required for efficient radiosensitization, it is still comparable to studies employing the methylating agent temozolomide, a clinically-employed radiosensitizer for adult glioblastomas^49^. Notably, **2** possesses a much lower radiosensitising effect than **1**; treatment with 40 μM **2** results an RER of 1.18 (Fig. 6a and Table S2). The formation of γH2AX is an early response to IR-induced DSB generation and may provide an indication of DNA damage levels^41^. As shown in Fig. 6b, cells pre-treated with **1** before 6 Gy irradiation showed γH2AX levels approximately two-fold greater compared to IR alone, indicating increased levels of DNA damage in the radiosensitised cells. Cells pre-treated with complex **2** demonstrated no such increase in γH2AX expression, where levels are comparable to cells mock-treated and exposed to 6 Gy IR. These findings indicate increased levels of DNA damage at an early time point accompany the enhanced radiation-induced cell killing in HeLa cells radiosensitised by **1**.

## Discussion

The isolation of small molecules that bind DNA and interfere with replication has been a major field of investigation, both in terms of generating therapeutic candidates to halt the growth of cancer and identifying pharmacological agents for the study of cellular DNA processes such as replication fork dynamics^2^,^3^,^4^,^29^. In the present study, we find the ruthenium-based complex **1** to intercalate DNA with high affinity and to immediately stall replication fork progression in human cells, resulting in the activation of DNA damage checkpoints and impeding cell-cycle progression. The growth of highly proliferative cancer cells is inhibited preferentially as a result. This work represents the first time replication fork stalling as a direct result of a pure metallo-intercalation binding modality has been demonstrated in human cells; a significant finding in translating the established cell-free DNA binding properties of RPC intercalators to biological function.

It is striking that the hydroxyl-substituted derivative complex **2** demonstrates no impact on DNA replication fork progression or DDR activation, despite demonstrating comparable cellular uptake properties to **1**, and instead impacts cell metabolic activity via autophagy induction. Also of note are distinct differences in the cellular response to **1** compared to topoisomerase inhibitors such as the organic intercalators mitoxantrone and doxorubicin, where DSB pathway activation, G2/M cell-cycle arrest and extensive apoptosis are observed^8^, indicating fork stalling by **1** is unlikely to occur via this mechanism. One potential explanation is provided by the viscosity data, which indicate that complex **1**, but importantly not complex **2**, induces lengthening and unwinding of duplex DNA to a greater extent than standard intercalation, indicating **1** to be a multi-intercalator^33^, and induces significant structural distortion to duplex DNA upon binding. Combined with the steric bulk of **1**, we propose DNA multi-intercalation by **1** in cells presents a physical block to replication, either by the resultant duplex distortion presenting a physical barrier to fork progression or a more complex binding interaction occurring at the fork resulting in stabilisation. As **2** does not demonstrate this behaviour, we conclude the unsubstituted PIP ligand, in combination with one or both dppz groups, to be required for this effect. These observations merit more detailed investigation into the precise nature of binding between the resolved enantiomers of **1** with DNA and further examination of a greater range of RPC intercalators on replication fork dynamics.

Mechanistically, our results indicate that **1** inhibits cell proliferation without strong activation of DSB damage signalling pathways or inducing high levels of apoptosis (or other cell death pathways), common cellular responses to DNA synthesis inhibitors^6^,^7^, topoisomerase poisons^8^ or reactive ruthenium-halide complexes^50^. Furthermore, we note several structurally-related RPCs have been shown to induce DNA-damage independent apoptosis in cancer cell lines, where a common finding is the induction of mitochondrial dysfunction and accompanying ROS-generation^16^,^17^,^18^,^19^,^20^,^21^. In clear contrast to these studies, we find **1** inhibits cancer cell growth by Chk1-mediated G1-S cell-cycle arrest, where prolonged Chk1 pathway activation is in agreement with activation of the replication checkpoint stabilising stalled replication forks^3^,^39^. It follows that if checkpoint function is abrogated, stalled or stabilised forks will collapse, resulting in higher DSB levels and triggering apoptosis: a concept we demonstrate by co-treating cells with **1** plus the Chk1 inhibitor CHIR-124. As **1** is predominantly cytostatic, checkpoint abrogation therefore facilitates a clear mechanistic change, whereby the cellular response of cell-cycle arrest is bypassed and cell death via apoptosis instead results. As cancer cells often possess high rates of proliferation *and* deficiencies in their DNA damage repair mechanisms, checkpoint abrogation employing specific DDR inhibitors in combination with DNA-damaging drugs is viewed as a current anti-cancer strategy and several DDR inhibitors are undergoing clinical trials^29^. However, work utilising checkpoint inhibitors in this fashion has primarily employed potent cytotoxic DSB-generating agents which activate the G2 DNA damage checkpoint such as topoisomerase inhibitors^45^,^51^ and gemcitabine^52^ in combination. This study, the first time an RPC intercalator has been combined with a DDR inhibitor to potentiate cytotoxicity, shows how a cytostatic replication inhibitor combined with G1-S/intra-S checkpoint abrogation can likewise achieve dramatic synergy in cancer cells; a finding that may have significance in achieving cancer-specific cell killing whilst avoiding the use of toxic and mutagenic agents.

Within cancer medicine, the damage to healthy tissue and severe side-effects that result from the use of highly cytotoxic molecules is a well-established limit within conventional chemotherapy^28^,^29^. In particular, combination therapy employing a DNA-damaging agent with external beam X-rays offers significant benefits compared to either treatment in isolation and is an established treatment regimen for a range of cancers, including cervical cancer^5^. To date, metal complexes that have been examined as radiosensitizers are either highly cytotoxic compounds that undergo ligand substitution reactions (e.g. Pt(II)- or Ru(II)-halides)^53^,^54^ or porphyrin-based^55^ and, somewhat surprisingly, there has only been one reported study examining an inert RPC in combination with external beam ionising radiation^56^. It is therefore highly significant that complex **1** demonstrates substantial capability as a radiosensitizer, where an enhancement in cell killing comparable to gemcitabine^48^ is observed. That complex **2** demonstrates a much lower radiosensitizing capacity indicates the impact of **1** on DNA replication to be the likely mechanism of sensitisation; a hypothesis supported by **1**-sensitised cells demonstrating increased levels of DNA damage at an early timepoint post-irradiation. As **1** generates a substantial increase in cell sensitivity to ionising radiation whilst possessing mild cytotoxicity would be predicted to be a substantial advantage in a therapeutic situation as off-target tissue toxicity by the sensitizer may be minimised.

In summary, we have shown that the ruthenium(II) polypyridyl complex [Ru(dppz)_2_(PIP)]^2+^, **1**, intercalates DNA and acts to stall replication fork progression in human cancer cells, activating DNA replicative stress signalling responses and preventing cell growth by cell-cycle deregulation. We demonstrate how **1** may be combined with a pathway-specific DDR inhibitor to achieve synergistic cell killing in cancer cells and show **1** functions as a radiosensitizer in combination with external beam ionising radiation. These discoveries indicate that ruthenium polypyridyl compounds are useful tools to probe the molecular consequences of DNA intercalation and also strengthen the case that this class of compounds merit further investigation as anticancer drugs, especially within combination therapy roles.

## Methods

### Reagents

All chemicals were purchased from Sigma-Aldrich, unless stated otherwise. Antibodies for cleaved caspase 3, p-Chk1(Ser345), p-BRCA1(Ser1524), p-Chk2(Thr68) and total Chk2 were purchased from Cell Signaling. Antibodies for total Chk1 were purchased from Santa Cruz and γ-H2AX (p-H2AX at Ser139) from Millipore. Anti-β-actin antibodies were purchased from AbCam. LC3 antibodies (Cell Signaling) were a generous gift from Dr C. Zois. Alexa Fluor 488- or 594-conjugated secondary antibodies were purchased from Life Technologies.

### Synthesis of 1 and 2

[Ru(dppz)_2_(PIP)]^2+^. **1**, and [Ru(dppz)_2_(HPIP)]^2+^, **2**, were prepared by an adaptation of a previously reported synthetic pathway^30^. Briefly, Ru(dppz)_2_(Cl)_2_ was prepared using an adaptation of the method described by Sullivan *et al*.^57^ and added to equivalent molar of PIP or *p*-HPIP (prepared as described by Liu *et al*.^30^) in dry ethylene glycol and refluxed for 4 hours under nitrogen atmosphere. Concentrated KPF_6_ solution was added once the mixture was cooled to room temperature. The solution was filtered, the precipitate washed with distilled water and dried *in vacuo*. The crude product was purified by column chromatography using alumina oxide where the solvent system was 1:1 mixture of acetonitrile and toluene. In each case, the reddish orange band was collected by rotary evaporation and dried *in vacuo*. Complex **1**: Mass (Yield): 0.128 g (51.8%). ^1^H NMR (CD_3_CN, δ ppm): 9.67 (d, 2H, *J* = 8.0 Hz), 9.61 (dd, 4H, *J* = 5.7, 8.0 Hz), 9.11 (d, 1H, *J* = 8.0 Hz), 8.45 (m, 4H), 8.40 (d, 2H, *J* = 8.0 Hz), 8.29 (d, 2H, *J* = 8.0 Hz), 8.13 (m, 4H), 8.01 (d, 2H, *J* = 4.6), 7.78 (m, 6H), 7.59 (dd, 2H, *J* = 3.5, 8.0 Hz), 7.49 (t, 1H, *J* = 8.0 Hz). Elemental analysis (as PF_6_ salt): Calcd: C, 52.77; H, 2.58; N, 13.43; Found: C, 52.98; H, 2.79; N, 13.21. ESI-MS, m/z (%): [M^+^-PF_6_^−^] = 962.2, 481.1 [M^2+^-2PF_6_^−^]. Complex **2**: Mass (Yield): 0.131 g (52.4%). ^1^H NMR (CD_3_CN, δ ppm): 9.67 (d, 2H, *J* = 8.0 Hz), 9.63 (t, 4H, *J* = 6.9), 9.10 (s, 1H), 8.45 (m, 4H), 8.34 (s, 2H), 8.29 (d, 6H), 8.11 (t, 4H, *J* = 3.4 Hz), 8.01 (m, 2H), 7.9 (s, 2H), 7.81 (m, 4H), 5.00 (s, 1H). Elemental analysis (as PF_6 _salt): Calcd: C, 52.10; H, 2.54; N, 13.26; Found: C, 52.52; H, 2.59; N, 13.27. ESI-MS, m/z: [M^+^+PF_6_^−^] = 1122.9, [M^+^] = 978.2.

### DNA binding

Calf thymus DNA, CT-DNA, (Sigma) was dissolved in NaCl (25 mm) and Tris (5 mm, pH 7.0) buffer and concentrations of determined spectroscopically (ε = 6600 dm^3^ mol^−1^cm^−1^ at 260 nm). Purity of DNA was verified by UV-Vis absorption, where A_260_/A_280_ > 1.9 indicated a protein-free sample. UV-Vis titrations and intrinsic binding constants were performed following the methods described by Liu *et al*.^30^. Viscosity experiments were performed as described in a recent publication^58^. Briefly, DNA was broken into an average of 150–200 base pair (bp) fragments by sonication. Viscosity measurements employed a DNA concentration of 4.19 × 10^−5 ^M where additions of the ligand to ≈50 μM/ bp DNA were made so that the values of r (r = [ligand]/[DNA]) were between 0 and 0.3. An equilibration time of 20 min was allowed before the flow times were recorded by Cannon–Manning semimicro viscometer (size 50) immersed in a thermostat bath (27 °C). Times were recorded in triplicate to within 0.1 second of each other and averaged values obtained.

### Cell culture

HeLa cervical cancer and primary human foreskin fibroblasts (HFF) cells were cultured in DMEM supplemented with 10% FBS and penicillin/streptomycin. MCF7 breast cancer cells were cultured in RPMI supplemented with 10% FBS and penicillin/streptomycin. HSAEC1-KT epithelial cells (ATCC) were cultured in Small Airway Epithelial Cell Growth Medium (Lonza) supplemented with the contents of the SAGM SingleQuot Kit. Cell lines were maintained at 37 °C in an atmosphere of 5% CO_2_ and routinely sub-cultured by Trypsin. Immortal cell lines were used at passage numbers 30 or lower and checked to be mycoplasma-free on a monthly basis. Stock solutions of **1** and **2** (20 mM) or CHIR-124 (10 mM) were prepared in DMSO before dilution in cell medium (final DMSO concentration = 0.5%). Cells treated with **1** or **2** were shielded from light to minimise phototoxicity. Cisplatin (2 mM) and mitoxantrone (5 mM) stock solutions were prepared in 100% PBS and each agent applied in DMSO-free media.

### MTT assay

Cells were seeded in 96 well plates at 20,000 cells/well, allowed to proliferate for 24 h before treatment as described in the main text. 0.5 mg ml^−1 ^MTT (thiazolyl blue tetrazolium bromide) dissolved in serum-free media was added for 60 minutes and the formazan product eluted using acidified isopropanol. The absorbance at 540 nm was quantified by plate reader (reference wavelength 650 nm). The metabolic activity of the cell population was determined as a percentage of a negative (solvent) control.

### Trypan Blue exclusion assay

HeLa cells were seeded in 6 well plates at 100,000 cells/well and allowed to proliferate for 24 h. Cell cultures were treated as stated, media containing detached/dead cells were removed and retained, adherent cells washed with PBS and detached with Trypsin. Adherent and detached cells for each sample were combined, concentrated via centrifugation and re-suspended in 0.5 ml PBS containing 0.04% Trypan Blue solution. Trypan Blue-negative and Trypan Blue-positive cells were counted by haemocytometer. A minimum of 200 cells were counted for each sample.

### Microscopy

Monolayers were grown in Ibidi μdishes (Thistle Scientific) or Lab-Tek II chamber slides (Thermo) and allowed to proliferate for 24 h. Cells were treated as stated in the main text, washed with PBS and fixed in paraformaldehyde (4%, 10 mins). For samples co-stained with DAPI (500 nM, 2 min) this was added after fixation and membrane-permeabilisation (0.1% Triton, 10 mins) steps. Epi-fluorescent images were obtained using a Nikon 90i upright widefield microscope and a x60 oil-immersion objective. An in-house filter cube comprising FITC excitation with TRITC emission was employed to detect MLCT emission of **1** and **2**. Confocal images were obtained using a Zeiss LSM 780 META inverted confocal microscope and x40 or x63 oil-immersion objectives. Microscopy images were processed using either Zeiss LSM Image Browser or ImageJ software.

### Sample preparation for ICP-MS

For whole cell extracts, HeLa cells were treated with 40 μM **1** or **2** for 24 h and detached with Trypsin. Samples were collected by centrifugation, washed in PBS and counted. Cells(5 × 10^5^) were then collected and lysed in RIPA buffer. For subcellular fractionation, 2 × 10^6^ HeLa cells were treated with 40 μM **1** or **2** for 24 h and subcellular fractionation performed using a Subcellular Protein Fractionation Kit for Cultured Cells (ThermoFisher Scientific), as described within the manufacturer’s manual. Separate cytoplasmic, membrane, nuclear soluble, chromatin-bound and cytoskeletal protein fractions were obtained. The nuclear soluble and chromatin-bound fractions combined into a single nuclear fraction while the cytoplasmic and cytoskeletal protein fractions were combined before analysis. Protein content for each fraction was determined using Pierce^TM^ BCA protein assay Kit (Thermo) employing the protein standard provided. Fractionated proteins were subjected to Western blot analysis using anti-α-tubulin (Sigma), anti-histone H2AZ (Abcam) and anti-β-integrin1 (Abcam) for cytoplasm/cytoskeleton, nuclear and membrane fractions respectively.

### ICP-MS analysis

Samples were microwave digested as described elsewhere^59^ and analysed by Perkin Elmer Elan 6100DRC ICP-MS. Calibration was achieved by external standards, diluted from a 1000 ppm Merck Certipur Ru standard (lot: HC42234247). A ruthenium standard (Merck multi element 10 ppm PGE standard, lot: A53740) was diluted and measured as a sample to verify the calibration. All blanks, standards and samples were spiked with an internal standard (1 ng/g Rh), so that any instrument drift could be normalised. All dilutions were made using 2% nitric acid prepared in deionised water. All data provided are elemental concentrations and measured from m/z (mass to charge ratio) 99 and 101. Untreated HeLa cell controls were included for analysis, however, the ruthenium content in these samples were beneath the detection limits of the instrument.

### DNA fibre assay

DNA fibre assays and immunofluorescent staining were performed as described elsewhere^60^, employing sequential CldU and IdU incubation times of 30 mins each. The second nucleotide (IdU) was incubated in the presence of **1**, **2** (40 μM) or a DMSO (0.5%) control. Fibres were visualised with a Leica DMRB microscope with a DFC360FX camera. Lengths of CldU- and IdU-labeled tracts were measured by ImageJ software. Statistical analysis was performed using GraphPad Prism software using unpaired t-test.

### Immunoblot analysis

Cells were incubated as stated, washed with cold PBS and lysed in RIPA (radioimmunoprecipitation assay) buffer containing protease inhibitors (10 μg/ml leupeptin, 2 μg/ml pepstatin, 50 μg/ml antipain, 2 μg/ml aprotinin, 20 μg/ml chyprostatin, 2 μg/ml benzamidine, 1 mM phenylmethanesulfonyl fluoride) and phosphatase inhibitors (50 mM NaF, 1 mM Na_3_VO_4 _and 20 mM β-glycerophosphate). Aliquots of cell lysates (5–20 μg total protein) were resolved by NuPAGE^®^ 4–12% Bis-Tris gels and LDS-PAGE, transferred onto nitrocellulose membrane and probed with primary antibodies in 5% BSA (bovine serum albumin) solutions. Reactions were visualised with a suitable secondary antibody conjugated with horseradish peroxidase (Thermo). Pierce ECL (Thermo) or WesternSurePREMIUM (Li-Cor) chemiluminescent substrates with X-ray development (Fuji medical film and Optimax 2010 processor) or digital analysis (LiCor C-Digit Blot Scanner) were used to visualise protein expression. Li-Cor Image Studio Lite software was used for densitometry data acquisition.

### Immunofluorescence

Cells were grown in Ibidi μ-dishes (Thistle Scientific) for 24 h and treated as stated. Samples were washed with cold PBS, fixed with paraformaldehyde (4%, 10 mins) and permeabilised with Triton (0.1%, 10 mins). Samples were blocked in 1% BSA in PBS-T (PBS with 0.1% Tween) for 1 h, before being probed with γ-H2AX antibodies (1/500 in 1% BSA, PBS-T, 1 h), washed in PBS-T (3 × 5 mins) and treated with Alexa Fluor 488- or 594-conjugated secondary antibodies (1/200, 1% BSA in PBS-T, 1 h). Plates were washed with PBS-T (3 × 5 mins), stained with DAPI (5 μg/ml, 1 min) and visualised by a Zeiss LSM 780 inverted confocal microscope as described above.

### Cell-cycle analysis

Cells were washed with PBS and detached using Trypsin. After centrifugation at 1000 rpm for 5 min, cells were fixed in cold 70% ethanol for 30 mins, centrifuged and re-suspended in PBS. Samples were stained using 10 μg/ml propidium iodide (PI) and 200 μg/ml RNAase A for 30 mins before FACS analysis using a Biosciences LSRII Flow Cytometer. A minimum of 10,000 cells were counted for each sample and data were processed using FloJo software. Technical comment: Initially, we observed MLCT emission from RPCs (e.g. cellular uptake and/or debris) in the PI channel; a low-intensity emission that can appear as a false “sub-G1” population in cell-cycle analyses. A higher PI concentration than standard was employed to remove this artefact.

### Irradiation and clonogenic survival assay

HeLa cells were pre-treated with DMSO, **1** or **2** for 20 h and irradiated using a ^137^Cs γ-irradiator (IBL637, CIS Bio Int.; dose rate = 0.809 Gy min^−1^). Solutions were removed 4 h after irradiation; cells detached using Trypsin and re-seeded in 6 well plates at a density of 1000 cells/well (in triplicate). Cells were incubated for 7–10 days after re-seeding to allow colony formation before being fixed with 10% methanol, 10% acetic acid and stained with 0.4% methylene blue. Colonies containing 50 cells or greater were counted using a Gelcount instrument and accompanying software (Oxford Optronix). Plating efficiencies were determined for each treatment condition and normalised to controls to provide the survival fraction. Resultant survival fraction (S.F.) versus radiation dose curves were fitted using a second order polynomial function (R^2^ values > 0.99).

All other experimental details are described in the Supplementary Information.

## Additional Information

**How to cite this article**: Gill, M. R. *et al*. A ruthenium polypyridyl intercalator stalls DNA replication forks, radiosensitizes human cancer cells and is enhanced by Chk1 inhibition. *Sci. Rep.* **6**, 31973; doi: 10.1038/srep31973 (2016).

## Supplementary Material

Supplementary Information

## Figures and Tables

**Table 1:** DNA binding properties of 1 and 2 and impact on cell proliferation.

			IC_50_ (μM)		
Compound	Log P	DNA Kb (M^−1^)	HeLa	MCF7	HFF
**1**	0.403	2.5 × 10^6^	38	28	>100
**2**	0.076	6.7 × 10^7^	14	12	>100
Cisplatin	−2.3	N/A	9	11	>100
MX	0.36	5 × 10^6^	3.0	3.6	4.2

Log P = octanol/water partition coefficient. K_b_ (M^−1^) determined from hypochromic shifts on addition of DNA. IC_50_ values were derived from 24 h proliferation assays.
