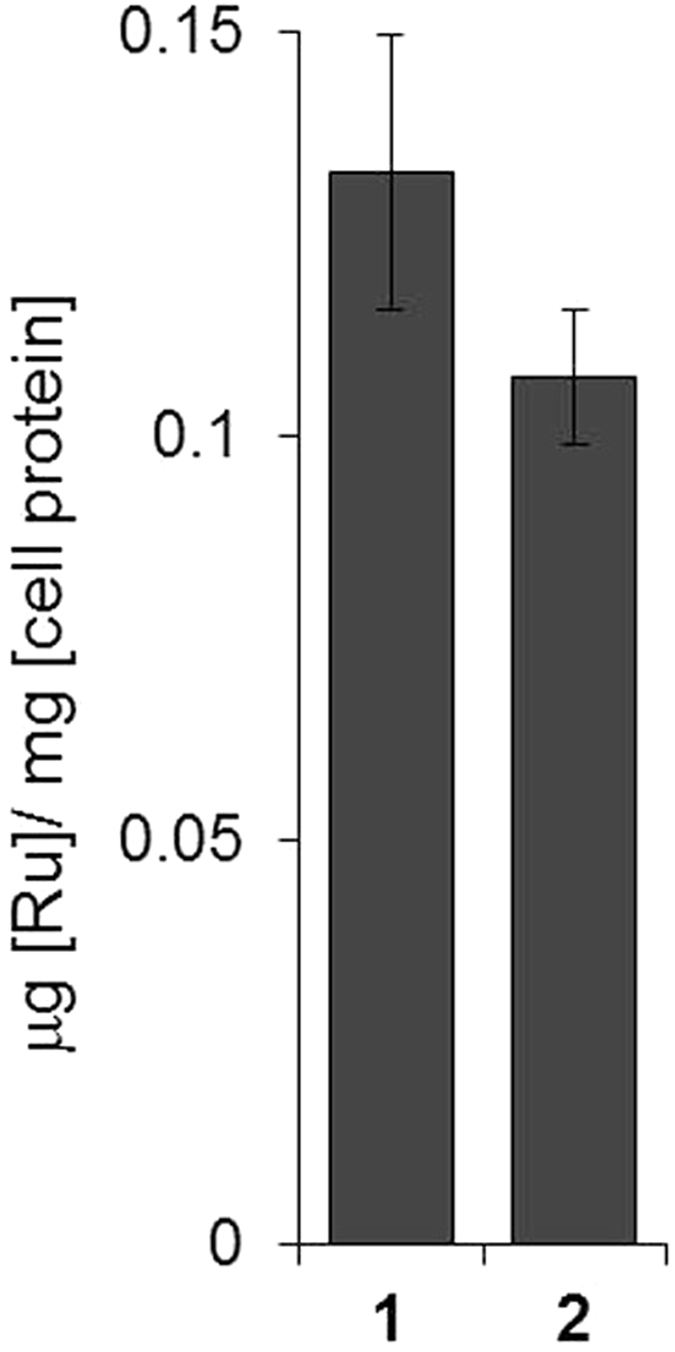# Corrigendum: A ruthenium polypyridyl intercalator stalls DNA replication forks, radiosensitizes human cancer cells and is enhanced by Chk1 inhibition

**DOI:** 10.1038/srep39363

**Published:** 2016-12-22

**Authors:** Martin R. Gill, Siti Norain Harun, Swagata Halder, Ramon A. Boghozian, Kristijan Ramadan, Haslina Ahmad, Katherine A. Vallis

Scientific Reports 6: Article number: 3197310.1038/srep31973; published online: 08 25 2016; updated: 12 22 2016

This Article contains an error in Figure 2c, where the y-axis ‘μg [Ru]/mg [cell protein]’ is incorrectly labelled as ‘ng [Ru]/mg [cell protein]’. The correct Figure 2c appears below as Figure 1.

## Figures and Tables

**Figure 1:**