# Supplementary material for: A ruthenium polypyridyl intercalator stalls DNA replication forks, radiosensitizes human cancer cells and is enhanced by Chk1 inhibition
Source: Sci Rep. 2016 Aug 25;6:31973. doi: 10.1038/srep31973 (PMC4997316; doi:10.1038/srep31973)
Supplement: Supplementary Information [file srep31973-s1.pdf]

Supplementary Information:

A ruthenium polypyridyl intercalator stalls DNA replication forks, radiosensitizes human cancer cells and is enhanced by Chk1 inhibition

Martin R. Gill[1], Siti Norain Harun[2], Swagata Halder[1], Ramon A. Boghozian[1], Kristijan Ramadan[1], Haslina Ahmad[2] and Katherine A. Vallis[1] *

1 CRUK/MRC Oxford Institute for Radiation Oncology, Department of Oncology, University of Oxford, Oxford, UK

2 Department of Chemistry, Faculty of Science, Universiti Putra Malaysia, Malaysia

* email: katherine.vallis@oncology.ox.ac.uk

Contents:

Supplementary Figures 1 – 14 (p2)

Supplementary Tables 1 and 2 (p13)

Supplementary Methods (p14)

Supplementary References (p15)

Supplementary Figures:

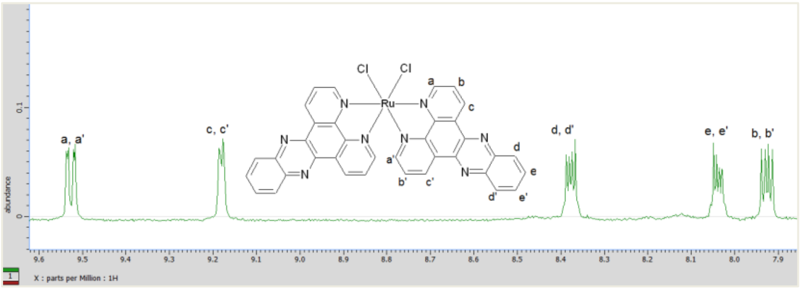

Figure S1. ^{1}H NMR spectrum of $Ru(dppz)_2Cl_2$ in C_2D_6OS.

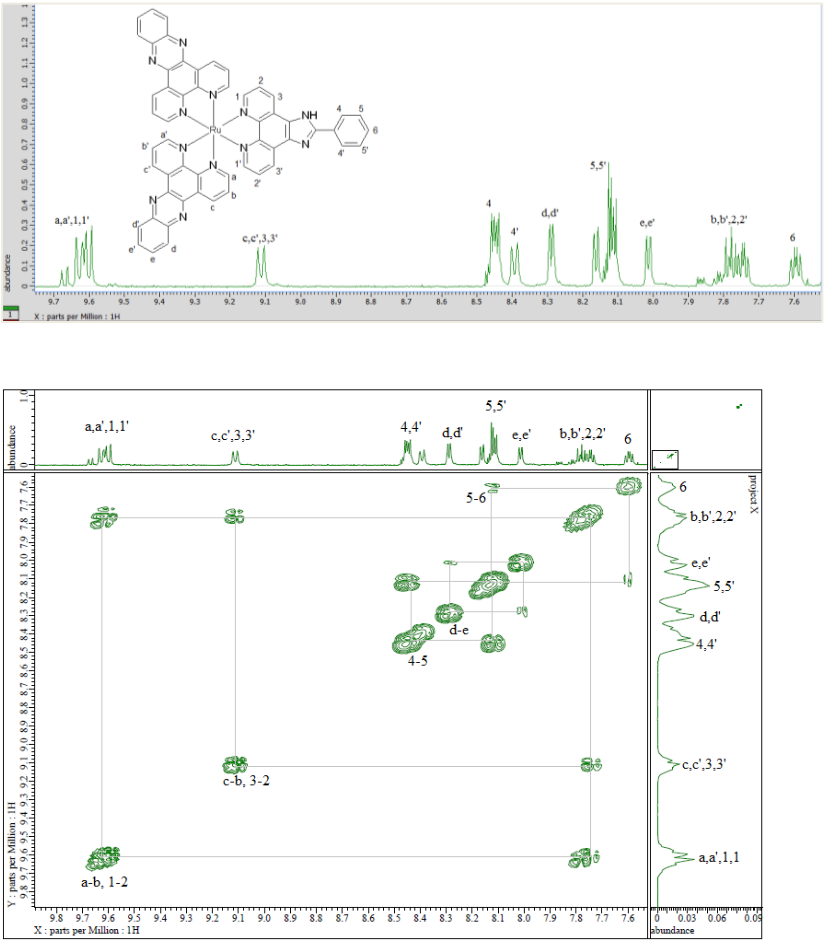

Figure S2. ^{1}H NMR (top) and 2D COSY ^{1}H NMR (bottom) of of $[Ru(dppz)_2PIP]^{2+}$ (complex **1**) in CD_3CN.

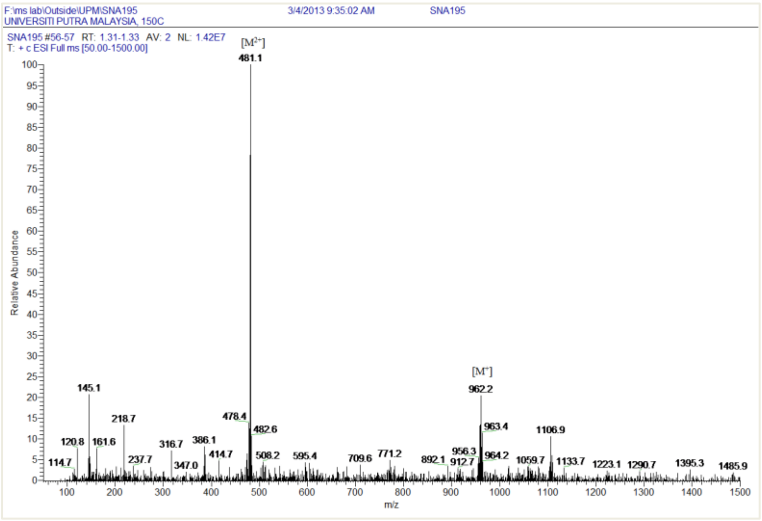

Figure S3. ESI mass spectrum of **1**.

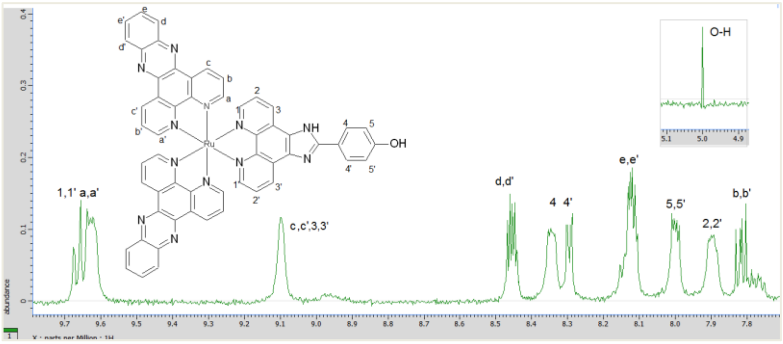

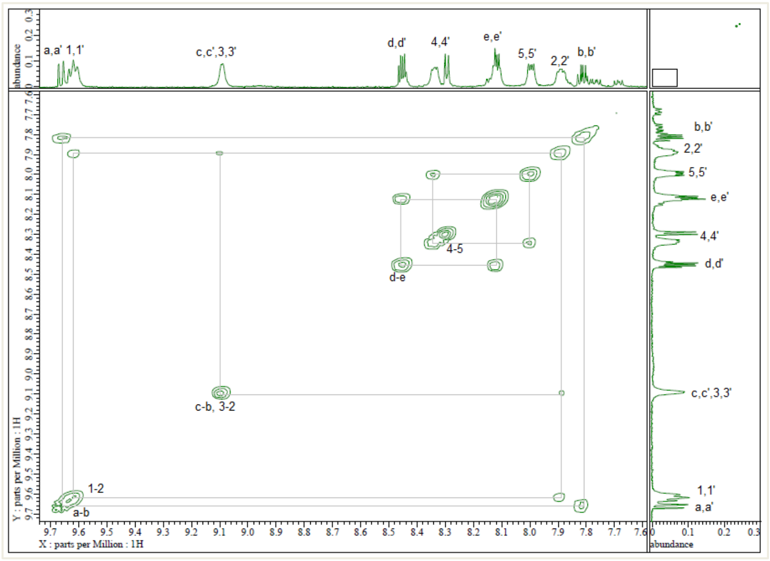

Figure S4. ^{1}H NMR of $[Ru(dppz)_2HPIP]^{2+}$ (complex **2**) (top) and 2D COSY ^{1}H NMR (bottom) in CD_3CN.

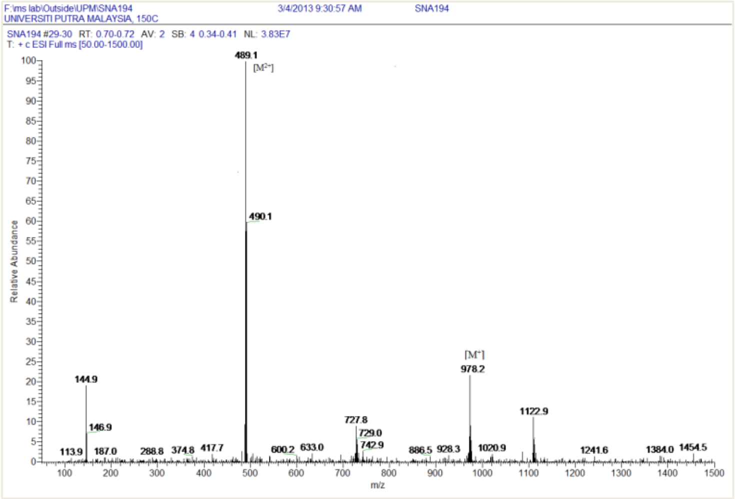

Figure S5. ESI mass spectrum of **2**.

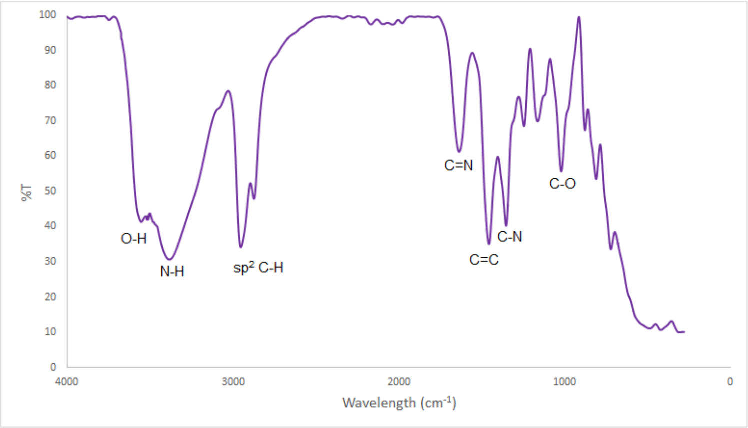

Figure S6. FT-IR spectrum of **2**.

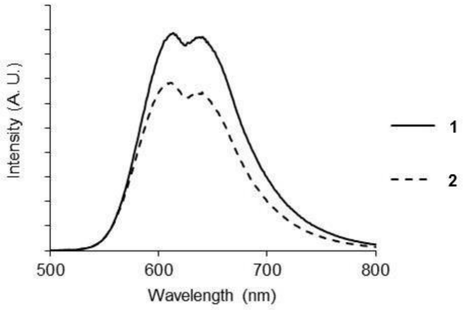

Figure S7. Luminescence emission spectra of 10 μM **1** and **2** in acetonitrile at room temperature (excitation wavelength = 460 nm).

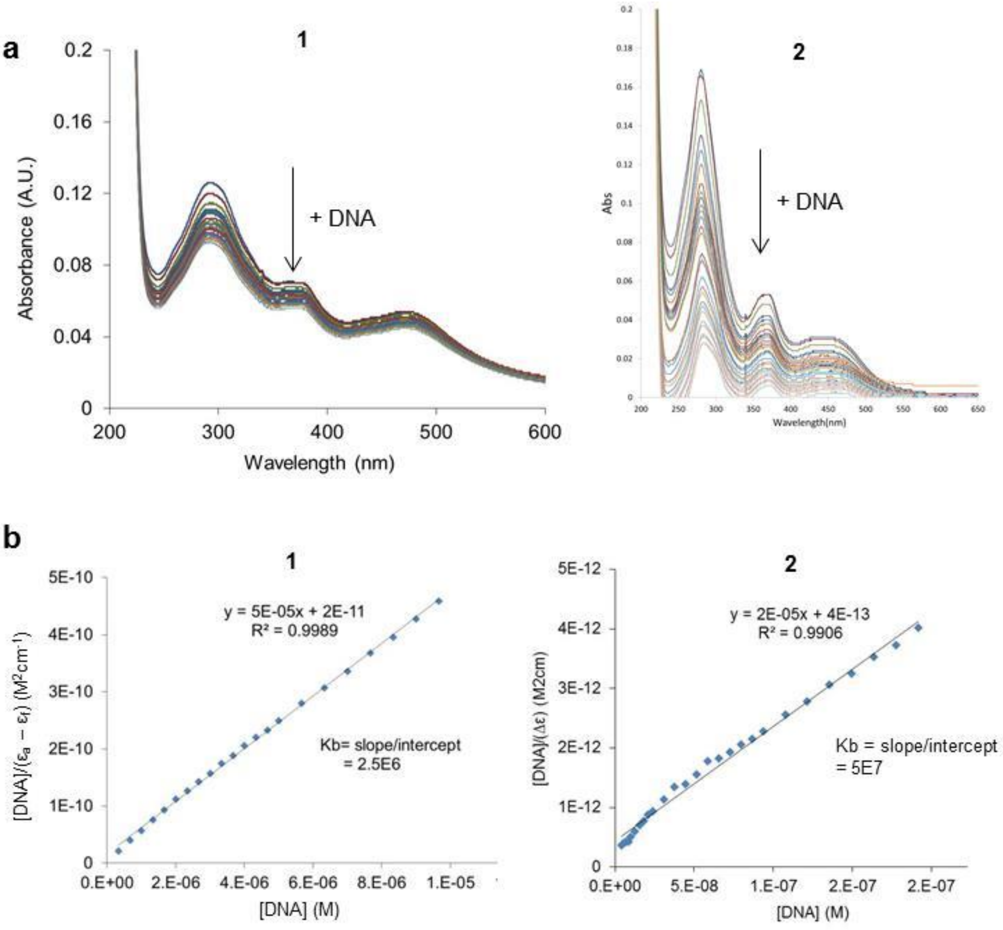

Figure S8. (a) Changes in UV-visible absorption spectrum of **1** or **2** with addition of DNA, indicating hypochromicity with increasing DNA concentration (buffer: 5 mM Tris-HCl, 25 mM NaCl, pH 7.2, 0.5 % DMSO). (b) Example fits of [DNA]/(ε_a - ε_f) versus [DNA] for hypochromic shifts of **1** and **2** with DNA. K_b is provided by the slope to intercept ratio. [DNA] = concentration of DNA in base pairs, ε_a and ε_f are apparent extinction coefficient for free complex and A_{obs}/[M] respectively.

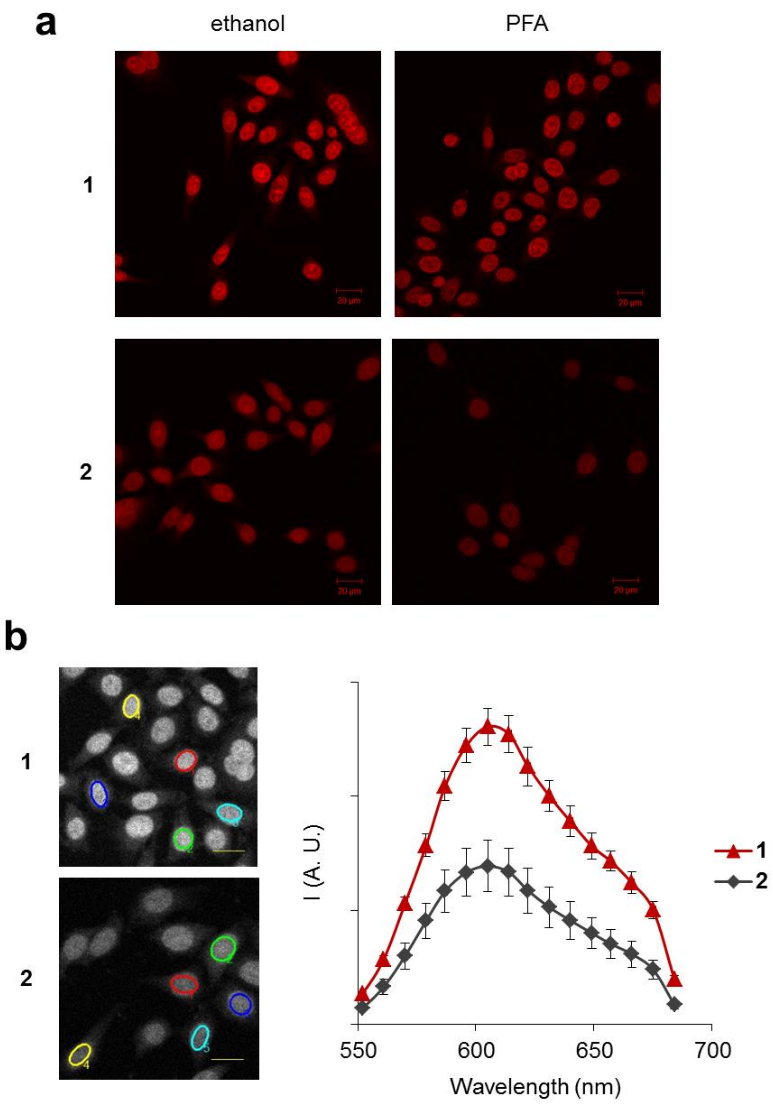

Figure S9. (a) Confocal micrographs of HeLa cells fixed with ethanol (70 %) or PFA (4% + 0.1 % Triton for membrane permeabilisation) showing cell nuclei stained by **1** or **2** (100 µM, 10 mins). (b) Emission profiles of **1** and **2**-stained HeLa cell nuclei. Data average of five nuclei stained (indicated by *, left hand images). Excitation wavelength = 488 nm. Identical microscope settings used for image acquisition.

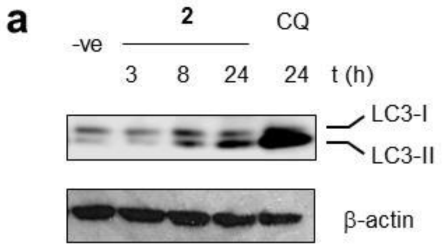

b

	-ve	2			CQ	
		3	8	24	24	t (h)
LC3-I	11300	13200	21200	13800	52300	
LC3-II	3820	7000	20500	25200	258000	
LC3-II/LC3-I	0.338	0.530	0.967	1.826	4.933	

Figure S10. (a) Immunoblotting analysis of lysates derived from HeLa cells treated with **2** (20 μM) for 3-24 h for increased levels of autophagy marker LC3-II (top blot, bottom band). β-actin levels were probed as a loading control. HeLa cells treated in parallel with chloroquine (CQ, 10 μM, 24 h) were employed as a positive control for LC3-II generation (see Supplementary Reference 1). (b) Densitometry of Western blots from (a). The increase in LC3-II/LC3-I ratio provides evidence of autophagy (see CQ data and supplementary reference 2).

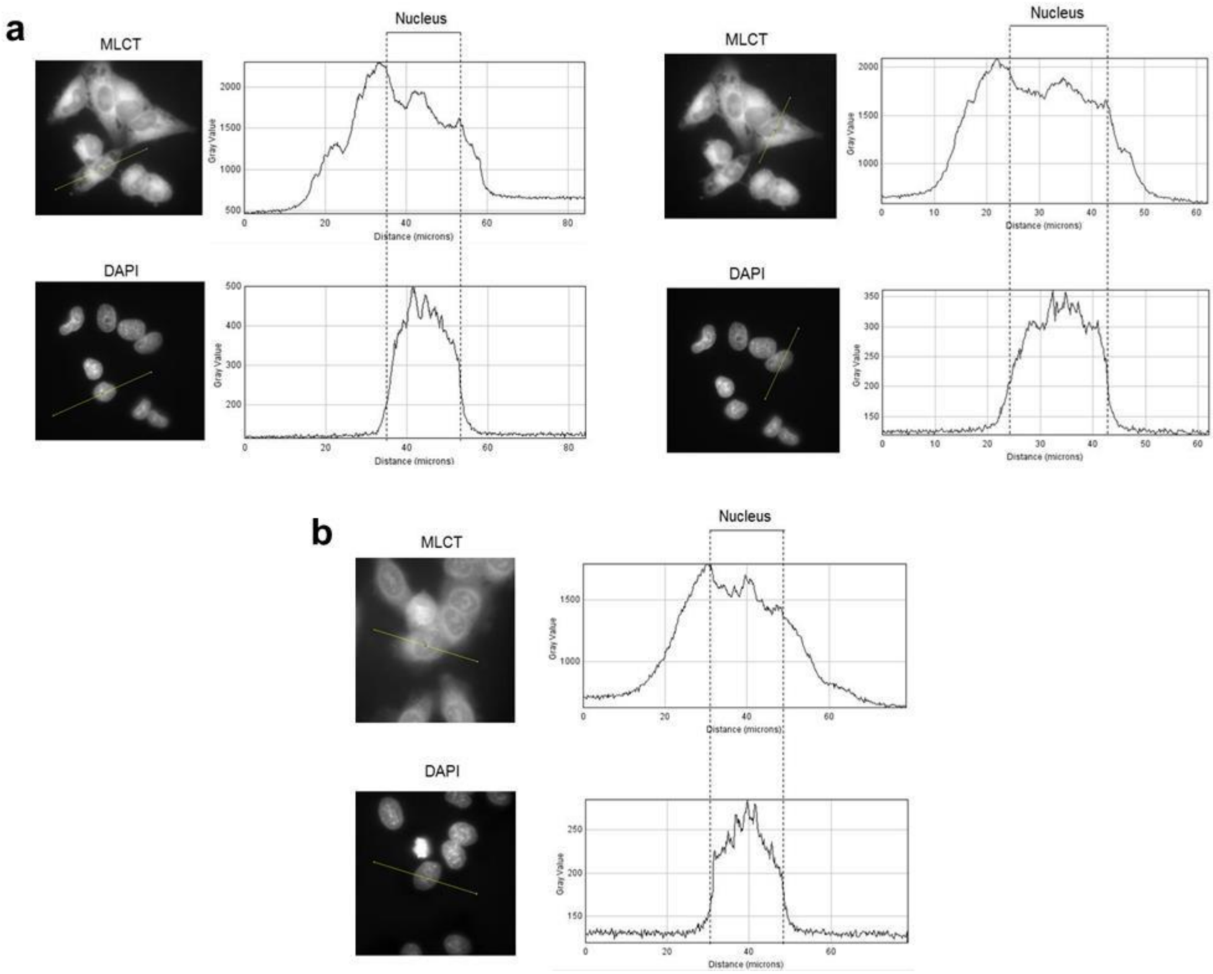

Figure S11. MLCT emission profiles of HeLa cells treated with **1** (a) or **2** (b) (40 μM, 24 h). DAPI emission profiles are included for comparison.

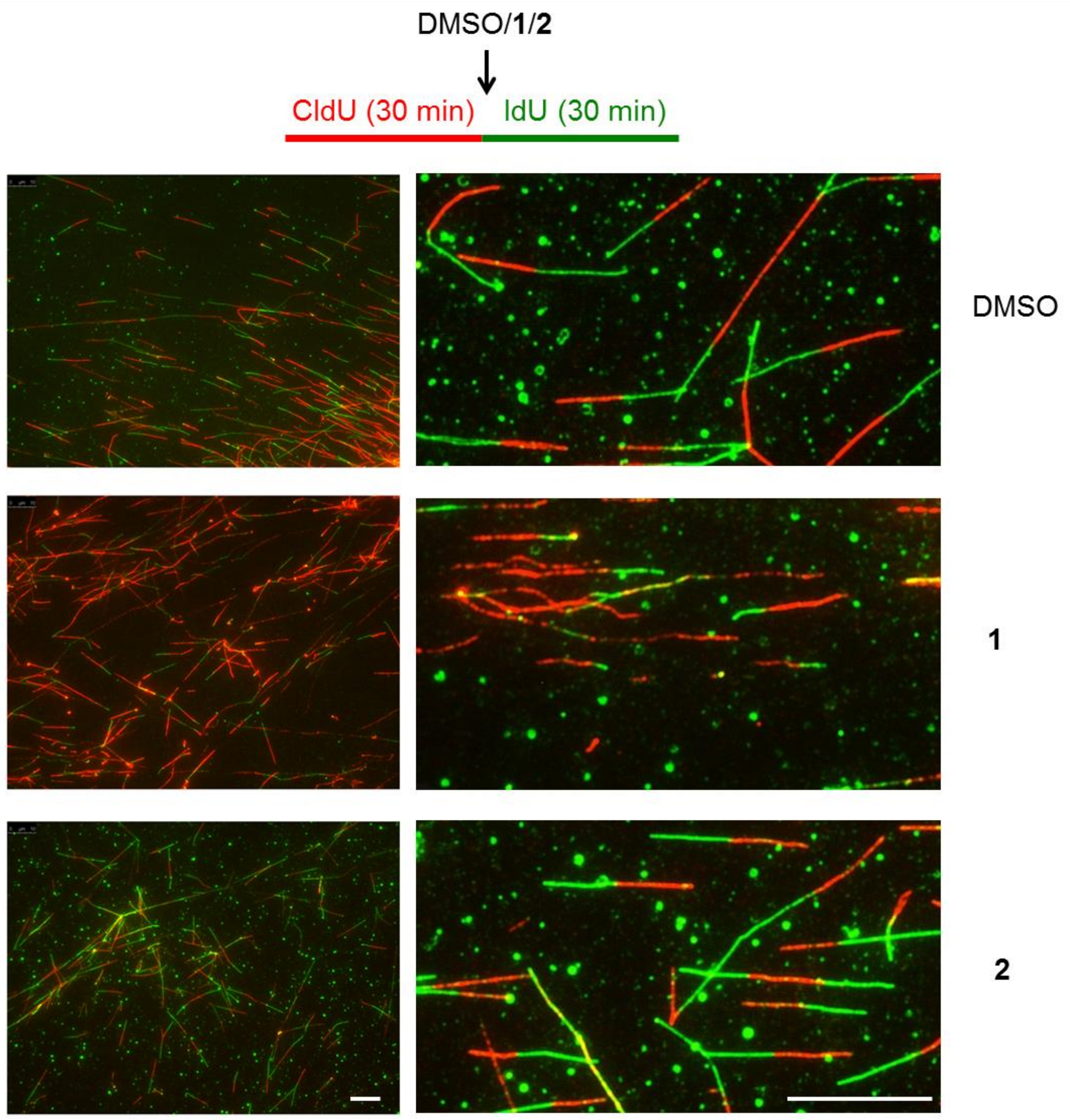

Figure S12. Representative DNA fibres labelled sequentially with CldU (red) and IdU (green) when the incorporation of the second synthetic nucleotide IdU (green) was in the absence or presence of **1** or **2** (40 µM). Scale bars = 10 µm. Note that IdU tracts (green) are shorter - or absent - with the addition of **1** compared to either DMSO (mock) or **2** treatment, indicating replication fork slowdown and stalling by complex **1**.

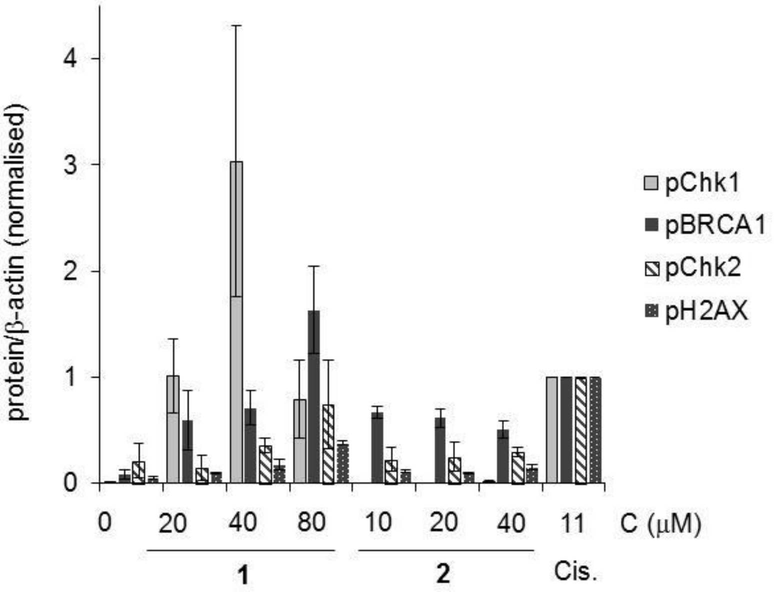

Figure S13. Densitometry of Western Blots of HeLa cell lysates treated with **1**, **2** or cisplatin (24 h). Phospho-DDR protein levels (pChk1, pBRCA1, pChk2, pH2AX) divided by protein loading controls (β-actin) and normalised to cisplatin results. Data average of two technical repeats +/- SD.

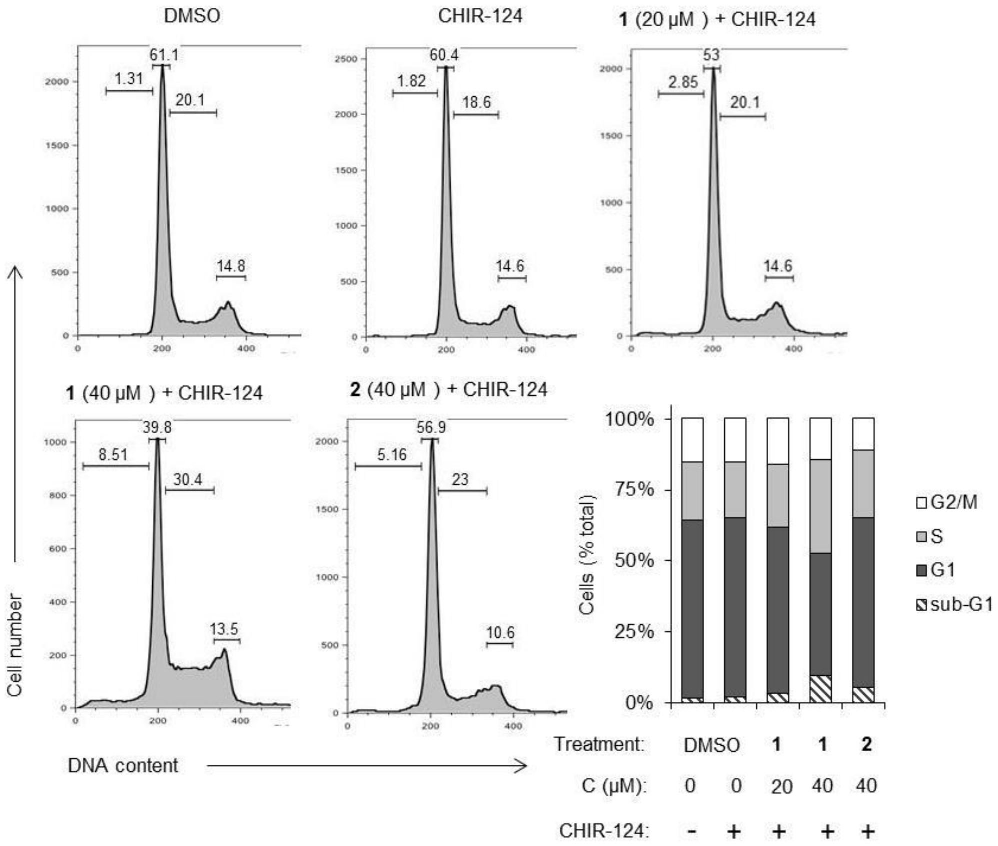

Figure S14. Cell-cycle distribution for HeLa cells incubated with **1** or **2** + CHIR-124 (500 nM) for 24 h. DNA content was quantified using propidium iodide (PI) and analysed by flow cytometry. Data summarised in bottom-right.

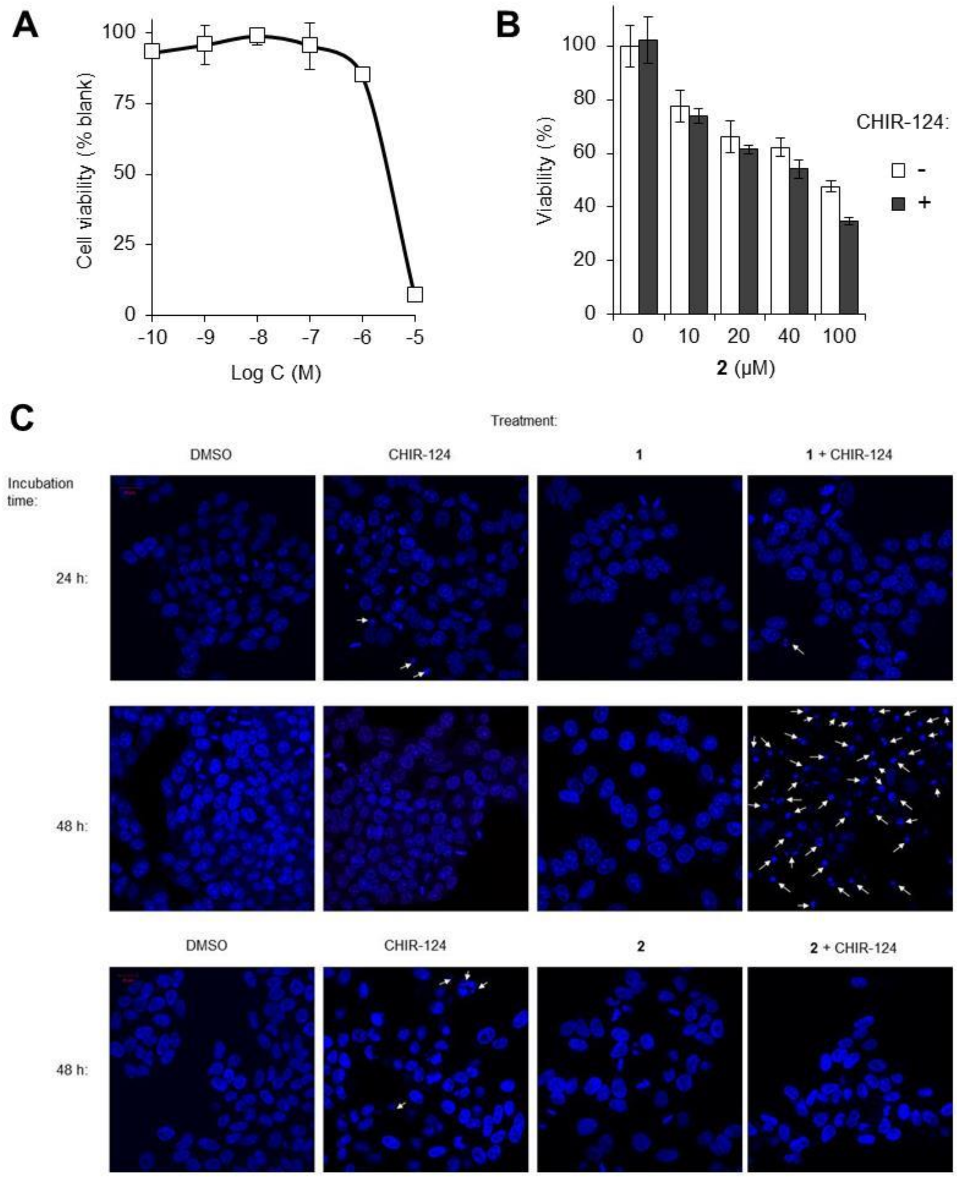

Figure S15. (a) Impact of Chk1 inhibitor CHIR-124 on cell viability of HeLa cells. Resultant cell viabilities assessed by MTT assay (in triplicate, +/- S.D.) after 48 h exposure. (b) HeLa cell viability after incubation with **2** in the absence or presence of Chk1 inhibitor CHIR-124 (500 nM) for 48 h constant exposure. (c) HeLa cells treated with either 40 µM **1** or **2** in the absence or presence of CHIR-124 (500 nM) for 24 h or 48 h. Late apoptotic nuclear morphology (pyknosis and/or karyorrhexis) indicated by arrows. Micrographs are representative of two independent experiments.

Supplementary Tables:

Table S1. UV-Vis absorbance data for **1** and **2**.*

**Complex**	**λ_{max} (nm)**	**ε ($M^{-1}cm^{-1}$)**	**Assignment**
**1**	458	5100	MLCT
	358	8470	π→π*
	280	37100	π→π*
**2**	458	10600	MLCT
	358	24580	π→π*
	280	86600	π→π*

*In acetonitrile at 293 K. MLCT = metal to ligand charge-transfer.

Table S2. Radiosensitization data for **1** and **2** in HeLa cells.

	LD_{10} (Gy)	DEF	Radiosensitivity (AUC)	RER
DMSO	6.7	-	3.83	-
**1**	4.5	1.49	2.59	1.48
**2**	5.9	1.14	3.39	1.13

Survival variables LD10 (10 % lethal dose) and radiosensitivity, which is expressed as the area under the survival curve (AUC), derived from linear regression analyses of survival curves (R^2 values ≥ 0.989 for all fits). Dose enhancement factor (DEF) = LD_{10} [without complex]/LD_{10} [with complex]. Radiation enhancement ratio (RER) = radiosensitivity [without complex]/radiosensitivity [with complex].

Supplementary Methods

Instrumentation

NMR spectra for ^{1}H and 2D-COSY were recorded on a JEOL ECX500 FT NMR spectrometer. Deuterated $CDCl_3$, DMSO or CD_3CN were used as solvents. Chemical shifts are relative to tetramethylsilane (TMS) as reference and are reported in ppm with coupling constants in Hertz (Hz). The multiplicities of peaks in ^{1}H NMR spectra were reported using abbreviation as follows: s - singlet, d – doublet, t – triplet, m – multiplet. Electrospray ionization mass spectra (ESI-MS) were measured on a Finnigan TSQ7000 mass spectrometer. Elemental analysis (C, H, N and O) were carried out using Leco CHNS-932 Elemental Analyzer. UV-Visible (UV-Vis) spectra were recorded on a Shimadzu H.U.V.1650 PC UV-Visible spectrophotometer for the wavelength range of 200-600 nm. Emission spectra were recorded over the range of 500-850 nm with excitation wavelength of 460 nm on a Shimadzu RF-5301 PC Spectrofluorophotometer.

Octanol/water partition coefficients

Octanol/water partition coefficients (Log P values) were calculated the "shake flask" method, where the concentration in each phase was determined by UV-Vis spectroscopy. Log P = log ([octanol]/[water]). The calculated log P value for mitoxantrone was determined using Molinspiration Chemoinformatics software (www. molinspiration.com).

Fixed tissue and fixed cell imaging

Sample preparation:

i) Frozen sections: MDA-MB-468 cell-line derived tumour xenografts and normal liver samples were a generous gift from Dr L. Song. Sections were fixed in 4 % paraformaldehyde (PFA) for 10 minutes, followed by permeabilisation in 1 % Triton/PBS for 10 minutes. Slides were incubated with **1** (0.1 – 5 mM, 1 h). Next, Sections were washed in PBS for 2 x 3 minutes and subsequently mounted with Vectashield® mounting medium (Vector Laboratories, Burlingame, CA) and coverslips. ii) Formalin-fixed paraffin-embedded sections: SQ20B cell-line pellets were deparaffinised using xylene (Sigma)

at room temperature for 2 x 3 minutes. Graded ethanol (Sigma) series comprising of 100 %, 95 %, 70 % and 50 % was used to rehydrate tumour sections, followed by permeabilisation in 1 % Triton/PBS for 10 minutes. Slides were incubated with **1** (5 mM, 1 h), washed with PBS (2 x 3 mins) and sections were mounted with mounting medium (Vector Laboratories) and coverslips. iii) Monolayer cultures: HeLa or MDA-MB-468 cells were fixed with paraformaldehyde (4 %, 10 mins) and additionally permabilised with Triton (0.1 %, 10 mins) before staining with **1** or **2** (100 µM, 1 h). Samples were washed with PBS and, where stated, co-stained with DAPI (500 nM, 2 min).

Microscopy:

Samples were visualised on Zeiss LSM 780 META inverted confocal microscope and x40 or x63 oil-immersion objectives, where **1** and **2** were excited with an Ar-ion laser at 488 nm and emission collected at 600-650 nm. Lambda stacking experiments employed collecting emission intensity data from 552-684 nm at intervals of 9 nm. DAPI was detected as standard.

Supplementary References

1. Ni, H.-M. *et al.* Dissecting the dynamic turnover of GFP-LC3 in the autolysosome. *Autophagy* **7**, 188-204 (2011)

2. Kabeya, Y. *et al.* LC3, GABARAP and GATE16 localize to autophagosomal membrane depending on form-II formation. *J. Cell Sci.* **117**, 2805-2812 (2004)
